# Building successful student-athlete coach relationships: examining coaching practices and commitment to the coach

**DOI:** 10.1186/2193-1801-3-383

**Published:** 2014-07-28

**Authors:** Davar Rezania, Robert Gurney

**Affiliations:** Department of Management, College of Business and Economics, University of Guelph, Guelph, Ontario N1G 2W1 Canada; School of business, MacEwan University, 10700 104 Ave NW, Edmonton, Alberta T5J 4S2 Canada

**Keywords:** Coach-athlete relationship, Commitment to the coach, Coaching teams, Intercollegiate sport, Coaching athletes, Social exchange, Leader member exchange, Information sharing, Teamwork, HRM and sports

## Abstract

In this study we utilized the concept of commitment to explain the impact of coaching practices on student-athlete’s behaviour. We examined the impact of commitment to the coach on the coaching outcome of in-role behaviour, and the influence of coaching practices, of information sharing, training, and encouraging teamwork, on the formation of relationships. We adopted measures from the organizational behaviour literature and surveyed student-athletes at two universities in Canada. The sample included data from 165 student-athletes from two universities. Results from structural equation modeling indicate support for the effect of coaching practices on commitment to the coach. Results also support the effect of commitment to the coach on the student-athletes’ role behaviour and performance. By showing that coaching practices impact commitment to the coach, and that commitment to the coach impacts student-athlete role behaviour and performance, the findings have important implications for a better understanding of the determinants of coaches’ and athletes’ performance. The managerial significance of this research rests in the insight provided into how coaching practices influence athlete’s behaviour through commitment to the coach. This study contributes to the literature on coach-athlete relationship within universities and colleges by applying the concept of commitment to the coach. This helps diversity research approaches to understanding coach-athlete relationships and extends prior research on commitment by looking at the context of the relationship between the student-athlete and their coach.

## Introduction

Athletics has become a prominent and central force in higher education in Canada and other countries. For universities or colleges involved, it is important to achieve desired performance goals. Thus, understanding factors that contribute to the success of student-athletes is essential for the management of interuniversity sport. In the sport management literature, factors such as Human Resources Management (HRM) practices and their contribution to the management of sport has received some research attention (Doherty 1998). Individual level HRM literature examines the relationship between characteristics of individual employees, their work perceptions and behavioural outcomes such as employee satisfaction, motivation, intention to leave, and citizenship behaviours (Rousseau and Greller 1994). Such studies have contributed to the development of HRM practices that help organizations manage expectation of their employees.

Managing student-athlete expectations is an important task for the universities as they sponsor and organize competitive sport in which student-athletes are participants. In addition, sport participation is an important educational element in the broader educational experience of students (Light and Dixon 2007). Much of the responsibility is placed on the coach to set the desired tone through policies and practices. To further understand the interpersonal dynamic between the coach and the player, it is important to understand the perceptions of the players with respect to effective coaching behaviours and practices (Garland and Barry 1988). It is important to understand how players interpret coaching practices and how those practices affect student-athlete performance (Shields et al. 1997).

**Figure 1:**
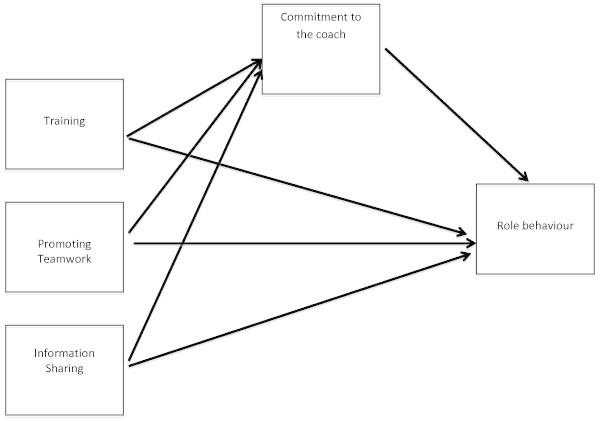
**Research model.**

Historically, coaching in sport has focused on developing athletes’ physical, technical and strategic skills by placing a great deal of time and energy on the technical and administrative aspects of coaching because these components were better defined and more controllable (Miller and Kerr 2002). Coach-athlete research has often focused on interpersonal dynamics between the coach and the athletes from a leadership approach (Salminen and Liukkonen 1996). More recently, research has evolved to investigate the effect of coaching behaviours on the coach-athlete relationships and the impact on outcomes, such as satisfaction (Poczwardowski et al. 2006).This paper provides a conceptual framework for examining the impact of coach-athlete relationships on coaching outcomes of role behaviour and performance, and the influence of coaching practices on building and maintaining the relationships. We use the concept of commitment as the construct against which to evaluate coach-athlete relationships. In terms of coaching behaviours, we consider coaching roles of training and development, information sharing, and encouraging participative decision-making. We explore the relationship between these constructs and student-athlete role-behaviour and performance. Figure 1 presents this research model.

This study is in line with Poczwardowski, Barott, and Jowett’s (2006) call to diversify research approaches to understanding coach-athlete relationships and contributes to the literature in two distinct but important ways. First, this study contributes to the literature on coach-athlete relationships by applying the concept of commitment to the coach. This extends the current research that considers how social exchange theory shapes the relationship between the coach and athlete. Second, this study extends prior research on commitment in educational settings by looking at the context of student-athletes and their relationship with their coach.

### Theoretical model and hypothesis development

Coach-athlete relationships have been defined as an interconnection of emotions, thoughts and behaviours (Jowett and Ntoumanis 2003). The coach-athlete relationship is intentionally developed through appreciation and respect for each other (Potrac et al. 2002), is both dynamic and complex (Jones and Wallace 2005), and requires discovering and fulfilling needs of both the coach and athlete (Jowett and Cockerill 2003). Numerous authors suggest that an effective coach-athlete relationship is necessary for a successful coaching outcome (Lafrenière et al. 2011; Shields et al. 1997). Factors that contribute to the coach-athlete relationship include, but are not limited to: planning and designing the coaching engagement, building and maintaining rapport, establishing and maintaining trust, building credibility (Mageau and Vallerand 2003, Rezania and Lingham 2009a)

Leader-member exchange (LMX) theory is often used to study a leader’s individual relationship interaction with their followers. LMX is a process theory of leadership that combines exchange and role theory (Graen and Uhl-Bien 1995), and is often used to account for development of high quality relationships between the leader and the subordinates (Bass 1990). The theory emphasizes that when leaders offer the opportunity for high-quality relationships, the performance of in-group members would increase (Graen et al. 1982). Developing a high quality relationship is a process that starts with the leader offering a membership in the in-group, followed by a period of “acquaintance” phase. Finally the “partner” phase is reached based on exchanges and development of trust and mutual respect (Graen and Uhl-Bien 1995). An outcome of high quality relationship is commitment (Meyer and Allen 1991).

In formulation of a theoretical model for the study of coach-athlete relationship, commitment provides a useful prototype. Meyer and Allen (1991) conceptualize commitment as a construct with three related dimensions. The affective dimension reflects the emotional aspect and encapsulates identification and involvement in the relationship; the continuance dimension relates to the perceived cost to leave the relationship, and finally; the normative dimension relates to the feeling of obligation to the relationship based on the congruence in values and norms.

Recent research into commitment has focused on investigating different targets of commitment within the organization. Commitment has been conceptualized to study a person’s relationship to another person in a social exchange (Cook and Emerson 1978), a person’s relationship to a group (Ellemers et al. 1997, Cropanzano and Mitchell 2005), a person’s relationship to an organization (Allen and Meyer 1990, Tzafrir and Enosh 2011), or person’s relationship to his/her supervisor (Stinglhamber and Vandenberghe 2003). Commitment to the supervisor has been studied as a factor that motivates citizenship behaviours (Redman and Snape 2005) or reduce employee turnover (Maertz et al. 2007).

Recently, the construct has been used to explain behavioural outcomes in educational contexts (McNally and Irving 2010). Considering the extant commitment literature in traditional workplace contexts, the educational institutions context, and the supervisory relationship context, it is reasonable to use the construct of commitment to the coach to evaluate coach-athlete relationship.

In line with the extant commitment literature, we conceptualize commitment to the coach as a strong belief in the goals and values (normative); a willingness to exert considerable effort on behalf of the coach (affective); and a strong desire to continue working (continuance) with the coach (Mowday et al. 1979; Sturges et al. 2005). In this manner, commitment refers to a sense of duty that a student-athlete feels to achieve the coach’s goals and to the willingness to do what is needed to perform well (Kline and Peters 1991). Such cohesion and identification with the coach emerges, for instance, when the coach properly demonstrates leadership in leading the team to success (Carron et al. 2002).

### Consequences of commitment to the coach

In the field of organization behaviour, commitment has been widely studied because it is predictive of work-related attitudes and behaviours such as motivation, engagement, retention, citizenship behaviours, or its relationship with the organizational effectiveness (Bishop et al. 2005, Williams and Anderson 1991). Meyer and Allen (1991) posit that when the affective, normative, or continuance commitment are high, behaviours will be more positive. The value of commitment to the organizational goals is recognized in the strategic approaches to human resources management that consider employee engagement as a means of enhancing performance (Green et al. 2006).

The relationship between a coach and athlete has similarities wit the relationship between a supervisor and an employee in an organizational setting. Similar to a supervisor, a coach has formal authority and may utilize both influence without authority and influence with authority when engaging with the athlete (Dansereau et al. 1975). This ability to employ both formal contractual and informal influence gives that the coach and the athlete some degrees of control over the type of relationship, or exchange that will exist between them. In the process of organizing their roles, the type of influence the coach employs affect the interpersonal exchange relationship between a coach and his/her athlete (Dansereau et al. 1975). The norm of reciprocity indicates that when the coach offers the athlete more latitude in things like decision making and signals the coach’s trust, respect, and support for the athlete, the athlete may then feel obligated to reciprocate with behaviours that would fulfill the coach’s expectation (Gouldner 1960). This belief in coach’s values and willingness to exert effort on his/her behalf is the basis for commitment to the coach. We therefore expect that commitment to the coach to be associated with student-athlete in-role behaviour.

H1. Commitment to the coach is positively associated with student-athlete in-role behaviour.

### Predictors of commitment to the coach

Training (T&D) student-athletes to attain high levels of performance is one of the most important responsibilities of a coach (Oliver et al. 2010). Training has the potential to draw a desired set of athlete’s attitudes and behaviours, and provides student-athletes the context to learn knowledge and skills for a specific purpose (Stein 2001). Training is an intentional activity to transfer the expertise, information, and also modify the attitude and behaviours aligned with the organizational goals (Brown and McCracken 2010).

In the organizational work settings, training is expected to influence job safety, self-importance, job satisfaction, and organizational commitment (Bartlett 2001). Training could be formal or informal. Formal training has a structured mode of delivery, is planned, and has a pre-set objective. Informal training, on the other hand, is less structured and is delivered on an ad-hoc basis. Whether formal or informal, training has a positive connection with organizational commitment (Owoyemi et al. 2011).

The effect of training on student-athlete’s role-behaviour and performance could be mediated by commitment. Student-athlete’s perception of the training she/he receives may contribute to the commitment to the coach. In addition, training may empower the student-athlete to work independently, participate in decision-making with other team members and work in the team. A coach who emphasizes training, may also emphasize enabling the student-athlete to take decisions in a decentralized manner and share with the team. Bishop et al. (2005) assert that the “level of support employees receive from an entity predicts the level of commitment they have for that same entity” (p. 175).

We therefore expect such training to be associated with commitment.

H2.a T&D is positively associated with commitment.

H2.b Commitment to the coach mediates the effect of T&D on student-athlete in-role behaviour.

Information sharing is another responsibility of the coach (Lyle 2002). Leadership literature indicates that follower performance, satisfaction, and retention are all influenced by relation with his/her immediate supervisor (Goleman et al. 2002). Student-athletes look to their coach for cues and information regarding what to do and how to do it. Coaching skills are firmly grounded in communication abilities including listening, feedback, and information sharing (Goleman et al. 2002). Communication is necessary for establishing and sustaining trust, and establishment of psychological contracts (Rousseau and Greller 1994). Information sharing reflects the extent to which coaches participate in the mentoring/coaching role to foster each student-athlete’s learning and development. At the heart of this facilitative coaching is an approachable communication style that fosters learning and development through clarifying expectations, providing relevant and up-to-date information, and enabling the student-athlete to obtain the relevant information (Sullivan and Gee 2007).

The effect of information sharing on student-athletes’ role-behaviour could be mediated by commitment to the coach. Student-athletes’ perception of the way the coach shares the necessary information may contribute to the commitment to the coach. In addition, information sharing should empower the student-athlete to participate in decentralized participative decision-making and work in the team (Kellett 1999). A coach, who emphasizes information sharing, may also emphasize enabling the student-athlete to participate in decision-making. We therefore expect information sharing to be associated with the commitment to the coach, and role behaviour.

H3.a Information sharing is positively associated with commitment to the coach.

H3.b Commitment to the coach mediates the effect of information on student-athlete in-role behaviour.

Leadership behaviours that lead to sharing power or giving more responsibility and autonomy to the followers have been the subject of many studies (Kirkman and Rosen 1999). Empowerment is considered an important mechanism for motivating and encouraging performance (Seibert et al. 2004). The effect of leadership behaviours on organizational commitment is indirectly affected by empowerment (Avolio et al. 2004). In this paper we consider promoting teamwork as coaches’ empowering behaviours that encourage student-athletes to participate in decentralized and participative decision-making and work as a team (Zimmerman 1990). This behaviour provides the student-athlete the skills and freedom to decide. We expect commitment to the coach to mediate the effect of promoting-teamwork on student athletes’ role behaviour

H4.a Promoting teamwork is positively associated with commitment.

H4.b Commitment to the coach mediates the effect of promoting teamwork on student-athlete in-role behaviour.

## Method

Our starting point was a questionnaire developed by Abdullah (2011) based on the previous work of Vlachos (2008), Green et al. (2006), Cook (1981), and Agho et al. (1992). This measure was developed to study the relationship between Human Resources activities such as training and development, information sharing, and decentralization on commitment and other outcome variables such as satisfaction, motivation, role-behaviour, and performance. As part of a project to study how social exchange influences the relationship between student-athletes and their coaches, we adopted Abdullah’s measure, but changed it to the context of the relationship between the coach and student-athletes. We checked for face validity by asking two faculty members and the Athletic Directors from participating universities to review the questionnaire and remove any ambiguous, vague and unfamiliar terms (Podsakoff et al. 2003). A seven item Likert scale was used (1 = strongly disagree, 7 = strongly agree) to capture the extent of agreement with each statement from each student athlete. In Table 1, items and their loading and cross loadings on the constructs are presented. The following statements are example of questions on the questionnaire:

**Table 1:** **Factor analysis**

	Commitment	Promoting teamwork	RoleB	T&D	Information sharing
willing to put in a great deal of effort	**0.74**	0.51	0.67	0.52	0.53
talk up this coach to my friends as a great coach	**0.86**	0.56	0.52	0.51	0.58
would accept assignments in order to keep playing for my coach	**0.79**	0.47	0.38	0.45	0.50
my personal values and the coach’s values are similar	**0.90**	0.59	0.57	0.48	0.66
proud to tell others that I play for my coach	**0.91**	0.60	0.54	0.53	0.62
My coach really inspires the very best in me	**0.91**	0.56	0.54	0.48	0.60
glad that I chose to be athlete under the direction of my coach	**0.92**	0.63	0.52	0.56	0.62
really care about the future of my role under my coach	**0.88**	0.59	0.58	0.51	0.59
generally feel informed by my coach about changes in my role	0.63	0.59	0.44	0.41	**0.89**
My coach keeps me informed and up-to-date	0.61	0.58	0.42	0.41	**0.87**
I know what is expected of me	0.52	0.50	0.45	0.45	**0.82**
I get adequate feedback from my coach	0.64	0.61	0.48	0.60	**0.86**
My coach communicates to me frequently and honestly	0.62	0.53	0.46	0.55	**0.90**
get the information I need to do well	0.49	0.55	0.49	0.41	**0.82**
adequately complete assigned tasks that my coach gives me	0.53	0.40	**0.83**	0.34	0.43
fulfill responsibilities specified by my coach	0.60	0.42	**0.89**	0.38	0.48
perform tasks that are expected of me, by my coach	0.57	0.45	**0.87**	0.39	0.44
meet formal performance expectations	0.56	0.47	**0.87**	0.36	0.49
engage in activities assigned by my coach,	0.53	0.35	**0.79**	0.39	0.43
don’t neglect aspects of a student-athlete that I am obligated to	0.40	0.39	**0.78**	0.32	0.38
I don’t fail to perform essential duties of a student-athlete	0.40	0.34	**0.73**	0.26	0.39
My coach systematically trains and develops my student-athletic abilities	0.48	0.47	0.38	**0.89**	0.45
My coach trains me to gain many skills and abilities	0.50	0.46	0.39	**0.90**	0.47
I receive from my coach the training I need to do well as a student-athlete	0.51	0.46	0.36	**0.89**	0.48
I receive the training and support from my coach that I need to perform well as a student-athlete	0.59	0.54	0.39	**0.90**	0.58
My coach encourages decentralized decision making	0.48	**0.79**	0.41	0.39	0.51
My coach encourages my team members to decide about sports specific performances and operational problems	0.58	**0.82**	0.37	0.42	0.52
We (team members) regularly work as a team to perform various tasks	0.48	**0.83**	0.31	0.42	0.48
My coach promotes teamwork	0.56	**0.80**	0.48	0.52	0.58

Bolded items indicate factor loadings on the respective constructs.

I am willing to put in a great deal of effort to help my coach build a successful team.

I talk up this coach to my friends as a great coach to play for.

I would accept almost any type of position assignment in order to keep playing for my coach.

I find that my personal values and the coach’s values are similar.

I am proud to tell others that I play for my coach.

My coach really inspires the very best in me in the way in which I perform.

I get adequate feedback from my coach.

My coach communicates to me frequently and honestly about issues affecting me as a student-athlete.

I can get the information I need to do well as a student-athlete.

The complete list of items is provided in Table 1.

As our objective was to study the construct in an educational setting, we chose a sample of student-athletes at two universities. Research assistants took the survey to the student-athletes and prior to the distribution of the survey, assured respondents of the anonymity and confidentiality of the study, explaining that there was no right or wrong answers, and that they should answer as honestly as possible. They also provided envelopes for returning the completed questionnaire

The questionnaire was distributed among 401 student-athletes from two universities. A total of 183 questionnaires were correctly completed and returned. The returned questionnaires revealed 71 females, 112 males; ages were between 18 and 23 years. The completed questionnaires reflected student-athletes from nine different sports, hockey, soccer, basketball, volleyball, curling, baseball, golf, swimming, and football. 165 returned questionnaires were filled by student-athletes participating in team sports. We used this set of questions for our analysis.

We utilized the Partial Least Squares (PLS) structural equation path modeling algorithm implemented in SmartPLS (Ringle et al. 2005). Because of our sample size, we could not use covariance-based structural equation modeling. However, our sample size met the minimum requirement of sample size for PLS analysis which in this study would be 30, ten times the largest number of structural paths directed at a particular construct in the inner path model (Hair et al. 2013). With a sample of 165, this requirement is met.

Table 1 presents the factor analysis of the constructs. All items loaded significantly (> .50) on their respective constructs. Furthermore, the cross loadings in Table 1 show that for each latent variable loading of each indicator is greater than its cross-loadings. This difference in loadings should be at least 0.10 (Gefen and Straub 2005). Our model meets this requirement, which implies indicator reliability. For discriminant validity, Table 2 reports composite reliability and Cronbach’s alpha. Composite reliability and Cronbach’s alpha values for all scales exceeded the minimum threshold level of .70, indicating the reliability of scales (Nunnally and Bernstein 1991). Table 2 also presents average variance extracted (AVE) criterion (Fornell and Larcker 1981). All latent constructs have AVE value greater than the minimum threshold value of .50, which implies convergent validity of constructs (Hair et al. 2013).

**Table 2:** **Reliability of indicators**

	AVE	Composite reliability	R Square	Cronbach’s alpha	Communality
Commitment	0.75	0.96	0.57	0.95	0.75
Promoting teamwork	0.65	0.88		0.82	0.65
RoleB	0.68	0.94	0.42	0.92	0.68
T&D	0.80	0.94		0.92	0.80
Information sharing	0.74	0.94		0.93	0.74

Table 3 presents the correlation among constructs. For checking discriminant validity of the measurement model we observe that in Table 3, the square root of AVE exceeds the correlations between the factors making each pair (Fornell and Larcker 1981). In addition, a correlation matrix also shows that none of the pairs of constructs correlate higher than 0.90, which indicates that common method bias is not a significant problem. In addition to this test, to control for common method bias, we had made sure that at least one of the variables were reverse coded. Furthermore, we ran a Harman’s one-factor test in SPSS on all the items in the model used to form the constructs. The first factor of the factor solution explained only 41% of the total variance. Overall these tests indicate that the common method bias is not a significant problem in this study.

**Table 3:** **Latent variable correlations**

	Commitment	Promoting teamwork	RoleB	T&D	Information sharing
Commitment	0.87				
Promoting teamwork	0.66	0.81			
RoleB	0.63	0.49	0.83		
T&D	0.59	0.54	0.43	0.89	
Information sharing	0.68	0.65	0.53	0.55	0.86

Note: diagonal cells are square root AVE, from Table 2.

## Results

We used PLS algorithm implemented in SmartPLS to estimate the paths between the constructs for testing the structural model. In addition, we performed the nonparametric bootstrapping procedure using 500 subsamples to evaluate the statistical significance of each path coefficient. Table 4 reports the results of this analysis. The PLS structural model is mainly evaluated by R^2^ of endogenous latent variable, and the path coefficients (Cohen 1988). The R^2^ values are presented in Table 2. The path coefficients (regression coefficients) are presented in Table 4. The effect size is presented in Table 5.

**Table 4:** **Path coefficients**

		Regression coefficient	T Statistics	Results
	Promoting teamwork - > RoleB	0.21	1.50	
	T&D - > RoleB	0.13	1.06	
	information sharing - > RoleB	0.32	2.61	
H1	Commitment - > RoleB	0.46	3.57	Supported
H2.a	T&D - > Commitment	0.22	2.10	Supported
H3.a	information sharing - > Commitment	0.37	3.04	Supported
H4.a	Promoting teamwork - > Commitment	0.30	2.73	Supported

**Table 5:** **Effect size**

	R Square- Included	R- square Excluded	f-squared	Effect size
information sharing - > commitment	0.574	0.506	0.16	Medium
T&D - > commitment	0.574	0.543	0.07	Small
Promoting teamwork - > commitment	0.574	0.528	0.16	Small
Commitment - > role behaviour	0.42	0.334	0.15	Medium

We tested the significance of a mediating effect of commitment to the coach on the relationship between coaching behaviours and role-behaviour, by using Baron and Kenny’s (1986) criteria. We build an additional model in which commitment to the coach was excluded. The mediating effect is presented in Table 6. We observe full mediation between information sharing and role-behaviour, and between encouraging teamwork and role-behaviour. The direct effect of training and development on role-behaviour is small and not significant. Baron & Kenny’s (1986) criteria is not met in this case.

**Table 6:** **Significant of mediation of commitment to the coach**

Hypothesis		Effect direct no Med	Effect indirect with Med	Iv - > Med Beta	Med - > DV Beta	IV - > Med SE	Med - > DV SE	Sobel’s test statistics	One tailed probability	Two tailed probability	Full or partial mediation
H2.b	T&D - > Role-beh	0.13									not significant
H3.b	Information sharing - > Role-Beh	0.32	0.15	0.37	0.46	0.12	0.13	2.32	0.10	0.02	full
H4.b	Promoting teamwork- > role-beh	0.22	0.08	0.30	0.46	0.11	0.13	2.17	0.02	0.03	full

By looking at Table 4 and path coefficients larger than 0.2, with T statistics higher than 1.96 (95% confidence interval) we observe that H1, H2.a, H3.a, H3.b, H4.a, and H4.b are supported, while H2.b, is not supported.

To control for gender and sport type, we added two constructs to our model with arrows to the outcome variable performance and run the PLS algorithm again. The paths had regression coefficients 0.089 and 0.031 respectively, indicating non-significant effects.

### Discussion and practical implications

We have examined how coaching behaviours of training and development, information sharing, and encouraging teamwork are related to one another; to commitment to the coach; and to student-athletes’ in-role behaviour. Several possible causal relationships were tested. In this manner, the prior research on the impact of coach-athlete relationship is extended by looking at how commitment to the coach mediates the effect of coaching behaviours on student-athlete role behaviour and performance.

A contribution of this study is to consider the case of intercollegiate sport at the universities and colleges, and conceptualize commitment to the coach in the same manner this concept is conceptualized in the human resources literature. This conceptualization enables us to explore the inter-relationship between coaching behaviours and their outcomes in terms of relationship with the coach. Most of the prior research has conceptualized commitment to the team or commitment to the sport (Casper et al. 2007).

First, we asked if commitment to the coach impacts role-behaviour. This study confirms the impact of athlete-coach relationships, measured against the construct of commitment, on student-athlete role behaviour. This is in line with the extant commitment literature in traditional workplace contexts and educational institutions that highlight the importance of commitment on behavioural outcomes.

Second, we asked if coaching behaviours such as training and development, information sharing, and encouraging teamwork impact commitment to the coach. The impact of information sharing on commitment is confirmed with a medium effect size. The impact of encouraging teamwork and training and development is confirmed, but the effect size is small. Thus, among the individuals in this study, it appears that student-athletes’ perceptions of the extent to which their coach trains them, and shares information and encourages them to work as a team is associated with the extent to which they feel committed to the coach. However, information sharing plays a much stronger role than training and development or encouraging teamwork. This indicates that coaches should pay significant attention to information sharing.

Third, we asked if commitment to the coach mediates the effect of coaching behaviours including training and development, information sharing, and encouraging teamwork on student-athletes’ role-behaviour. The results indicate that commitment to the coach mediates the effect of information sharing on role-bahaviour. When commitment to the coach is present, the direct effect of information sharing on role-behaviour is not significantly different than zero. The same is true for the relationship between encouraging teamwork and role-behaviour. Commitment to the coach is therefore a significant factor in explaining the importance of the coach-athlete relationship for athlete performance.

Our study contributes to the current literature in two ways. First, it contributes to the literature on coach-athlete relationship by applying the concept of commitment to the coach. This helps diversify research approaches to understanding coach-athlete relationships. This responds to the call by Poczwardowski et al. (2006) to extend the current research on coach-athlete relationships by considering how social exchange shapes the relationship between the coach and athlete.

Second, this study extends prior research on commitment by looking at the context of student-athletes and their relationship with their coach. Most of the prior research has focused on the commitment between an organization and its employees. As noted in the introduction of this study, the HRM literature reports a strong relationship between commitment and behavioural outcomes. The findings reinforce previous work in the HRM field and extend existing theories to the context of coaching athletic teams. Consistent with the premises of social exchange theory, it appears that team members (student-athletes) who feel committed to the coach are more likely to be willing to do more than is required and perform well.

Given that very little was found in the literature about the role of commitment to the coach in the context of coaching student-athletes, the presented results are encouraging and have important implications for developing coaching practices that lead to commitment to the coach. The results of this study indicate the importance of three coaching practices that lead to commitment, and the willingness to do more. It also indicates that among these three practices, information sharing plays a more important role than training and development or encouraging teamwork.

As Jowett and Cockerill (2003), suggest, the coach-athlete relationship is intentionally developed through appreciation and respect for each other, is both dynamic and complex, and requires discovering and fulfilling needs of both the coach and the athlete. Our study indicates that adopting practices that focus on training and development, sharing of information, and encouraging teamwork can contribute to the development of high quality relationships between the coach and the athlete, which in turn results in the athlete’s willingness to do more. Universities should therefor adopt policies and procedures that enable effective coaching practices. Such practices have been advocated by the HRM literature. Furthermore, HRM is able to provide guidelines about those organizational design elements such as recruitment, training, and job design that directly affect the performance of coaches. In this respect HRM is one of the crucial elements in managing the expectations of student-athletes.

The study highlights the ability of coaches to develop committed athletes. Athletic directors and sport manages should train and enable coaches to enhance their interaction with the athletes in order to provide them with opportunities to share information. The student-athlete should generally feel well informed by the coach about changes that affect them or about important issues concerning their role as a student-athlete. They should also have a complete understanding of what is expected of them in the student-athlete position and receive adequate and honest feedback. Student-athletes should be effectively facilitated to the information and resources needed to perform to their best abilities as a student-athlete. The universities should provide the required infrastructure and enable the coaches to reach these objectives.

As a teacher, a coach should be able to focus on providing student athletes with training on key skills and abilities. The training should be relevant and meet the needs of their role as a student-athlete and help them to perform well in that position. The coach should enable and empower the student-athlete to make decisions about sport specific issues. This will enable the student-athlete to perform effectively in the team.

The managerial significance of this research rests in the insight provided into how coaching practices influence athlete’s behaviour through commitment to the coach. Managers can influence those practices by institutionalizing best practices.

### Limitation and proposals for further studied

There are areas that need to be addressed in future research. First, our findings are limited in terms of the sample. We only surveyed participants from two universities in one province in Canada. The advantage to this was that the rules and policies around admission and compensation were similar in both universities. We realize that further studies should include teams from more universities across the country.

Further research could investigate the impact of other coaching behaviours or how student athlete’s personality traits affect commitment to the coach. For example, future studies could consider the effect of coaches’ leadership style or competencies. Such studies would help to better define what contribute to student-athlete role-behaviour and performance. Other studies could look at the effectiveness of a constructivist approach to information sharing and team coaching (e.g. Rezania and Lingham 2009b), for the development of commitment to the coach.

With reference to business-management perspectives, we have become very aware over the past decades that sport is a business. Exploring business-management concepts and theories through applications to sport and coach-athlete relationships appear to have focused on leadership and interpersonal behaviours. This study brings concepts of coaching studied in workplace environments (employer-employee relationships), as a means to investigate applications in the coach-athlete relationships in the context of intercollegiate sport. The results of this study will hopefully inspire others to duplicate and/or modify our methods of inquiry, as a means to better understand relationship constructs of coaching, in exploring coach-athlete relationships in a variety of sport environments.
